# Supplementary material for: Complex‐centric proteome profiling by SEC‐SWATH‐MS
Source: Mol Syst Biol. 2019 Jan 14;15(1):e8438. doi: 10.15252/msb.20188438 (PMC6346213; doi:10.15252/msb.20188438)
Supplement: Supplementary file 8 — Dataset EV7 [file MSB-15-e8438-s008.zip › feature_plots_string/P51809.pdf]

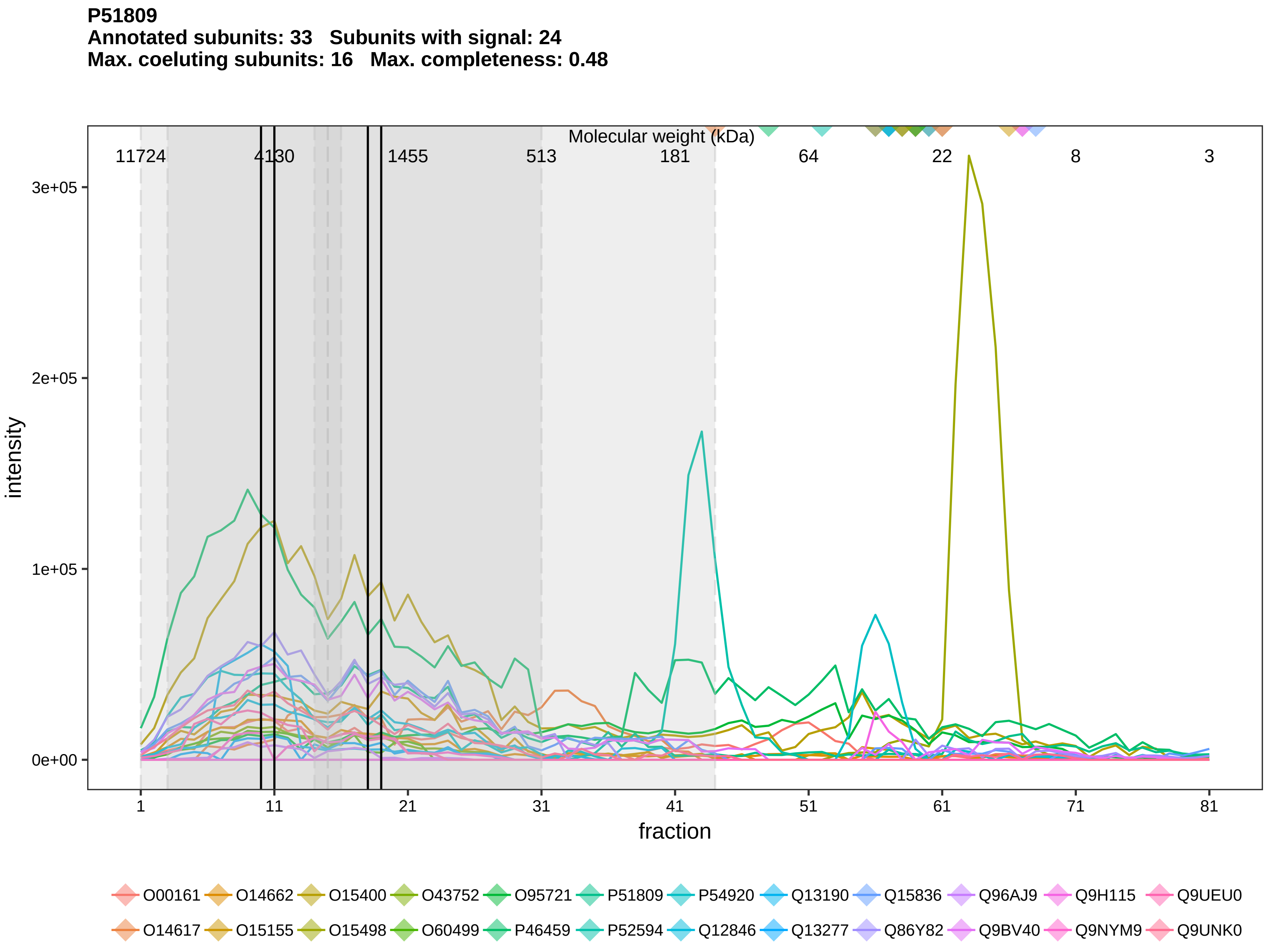
P51809
Annotated subunits: 33 Subunits with signal: 24
Max. coeluting subunits: 16 Max. completeness: 0.48
Molecular weight (kDa)
11724
4130
1455
513
181
64
22
8
3
3e+05
2e+05
1e+05
0e+00
intensity
1
11
21
31
41
51
61
71
81
fraction
O00161
O14662
O15400
O43752
O95721
P51809
P54920
Q13190
Q15836
Q96AJ9
Q9H115
Q9UEU0
O14617
O15155
O15498
O60499
P46459
P52594
Q12846
Q13277
Q86Y82
Q9BV40
Q9NYM9
Q9UNK0
